# Satisfactory patient-reported outcomes at 5 years following primary repair with suture tape augmentation for proximal anterior cruciate ligament tears

**DOI:** 10.1007/s00167-021-06485-z

**Published:** 2021-02-13

**Authors:** Graeme P. Hopper, Joanna M. S. Aithie, Joanne M. Jenkins, William T. Wilson, Gordon M. Mackay

**Affiliations:** 1 College of Medical, Veterinary and Life Sciences, University of Glasgow, University Avenue, Glasgow, G12 8QQ Scotland, UK; 2 NHS Greater Glasgow & Clyde, Glasgow, Scotland, UK; 3 University of Strathclyde, Glasgow, Scotland, UK; 4 University of Stirling, Stirling, UK

**Keywords:** Knee, ACL, ACL rupture, ACL repair

## Abstract

**Purpose:**

An enhanced understanding of anterior cruciate ligament (ACL) healing and advancements in arthroscopic instrumentation has resulted in a renewed interest in ACL repair. Augmentation of a ligament repair with suture tape reinforces the ligament and acts as a secondary stabilizer. This study assesses the 5-year patient-reported outcomes of primary repair with suture tape augmentation for proximal ACL tears.

**Methods:**

Thirty-seven consecutive patients undergoing ACL repair with suture tape augmentation for an acute proximal rupture were prospectively followed up for a minimum of 5 years. Patients with midsubstance and distal ruptures, poor ACL tissue quality, retracted ACL remnants and multiligament injuries were excluded. Patient-reported outcome measures were collated using the Knee Injury and Osteoarthritis Outcomes Score (KOOS), Visual Analogue Pain Scale (VAS-pain), Veterans RAND 12-Item Health Survey (VR-12) and the Marx Activity Scale. Patients with a re-rupture were identified.

**Results:**

Three patients were lost to follow-up leaving 34 patients in the final analysis (91.9%). The mean KOOS at 5 years was 88.5 (SD 13.8) which improved significantly from 48.7 (SD 18.3) preoperatively (*p* < 0.01). The VAS score improved from 2.3 (SD 1.7) to 1.0 (SD 1.5) and the VR-12 score improved from 35.9 (SD 10.3) to 52.4 (SD 5.9) at 5 years (*p* < 0.01). However, the Marx activity scale decreased from 12.4 (SD 3.4) pre-injury to 7.3 (SD 5.2) at 5 years (*p* = 0.02). Six patients had a re-rupture (17.6%) and have since undergone a conventional ACL reconstruction for their revision surgery with no issues since then. These patients were found to be younger and have higher initial Marx activity scores than the rest of the cohort (*p* < 0.05).

**Conclusion:**

Primary repair with suture tape augmentation for proximal ACL tears demonstrates satisfactory outcomes in 28 patients (82.4%) at 5-year follow-up. Six patients sustained a re-rupture and have no ongoing problems following treatment with a conventional ACL reconstruction. These patients were significantly younger and had higher initial Marx activity scores.

**Level of evidence:**

Level IV.

## Introduction

Primary repair of the ACL was often the treatment for ACL ruptures in the 1970s and 1980s [16, 42, 48]. However, failure rates of 25–53% were described at mid-term follow-up [15, 18, 33] and ACL reconstruction became the gold standard treatment in the 1990s [5, 14]. Reconstruction remains the gold standard today, despite a number of associated problems including graft harvest morbidity and graft failure [1, 4, 28–30, 32, 41, 47, 49].

Recent improvements in arthroscopic instrumentation, suture materials, imaging and rehabilitation protocols as well as an advanced understanding of ACL healing has led to renewed interest in primary repair [34]. Better outcomes have been demonstrated with primary repair of the ACL for selected patients with a proximal ACL rupture when compared to historic techniques [24, 26]. This avoids donor site morbidity and in the event of revision surgery, a routine ACL reconstruction can be performed. Primary repair means the native ACL is spared thereby retaining the proprioceptive fibres of the ACL which could be important for functional recovery. It is thought that the loss of these fibres can lead to a lack of confidence in the knee, which is a common complaint of patients following ACL reconstruction, despite restoration of joint laxity [6, 8, 20].

This study describes the 5-year outcomes of anterior cruciate ligament repair with suture tape augmentation. As far as we are aware, this is the first study with this length of minimum follow-up following this procedure. The hypothesis of this study was that the patient-reported outcome measures would be satisfactory 5 years postoperatively, with fewer failures than the 25–53% described in historic literature. This theory is through the addition of suture tape augmentation, retaining the proprioceptive fibres of the ACL and the avoidance of donor site morbidity [15, 18, 33].

## Materials and methods

This study was granted institutional review board approval by the University of Strathclyde (UEC19/24). Between 2011 and 2014, 37 patients with an acute proximal ACL rupture who underwent ACL repair with suture tape augmentation within 3 months of injury were included in this study. These patients were prospectively followed up for a minimum of 5 years postoperatively. Patients with midsubstance and distal ACL ruptures or retracted ACL remnants in this timeframe underwent a standard ACL reconstruction and were excluded from this study. Patients with multiligament knee injuries and chronic ruptures were excluded. Three patients were lost to follow-up leaving 34 patients in the final analysis (91.9%).

Mean follow-up was 68.0 (± 6.0) months (range, 60–89 months). The mean age at the time of surgery was 37.8 (± 15.5) years (range, 13–60). Eighteen patients were male and sixteen patients were female.

### Surgical technique

The ACL is probed to assess its suitability for primary repair. Proximal ruptures of the ACL are repaired with suture tape augmentation. A proximal tear was defined as having a long enough distal remnant for reattachment to the femoral footprint which equates to type I and type II tears in the modified Sherman classification described by Van der List et al. [46].

The ACL remnant is left intact and a standard tibial ACL guide is placed at the centre of the ACL footprint. A small skin incision is made above the pes anserinus and a 3.5-mm tibial tunnel is drilled. A suture is passed through the midsubstance of the ACL stump using a suture passer and retracted through the medial portal, forming a lasso around the distal ACL stump. The femoral attachment is then identified, microfracturing is performed and a 3.5-mm femoral tunnel is drilled. A femoral button loaded with suture tape is subsequently transported proximally through the tibial tunnel, the centre of the ACL and the femoral tunnel. The suture tape is fixed distally, just below the tibial tunnel, using a 4.75 mm anchor loaded with both ends of the suture tape.

Patients are allowed to fully weight bear with crutches as required during the first 2 weeks and physical therapy focuses on early range of movement, muscle control and restoration of function. No external bracing is required. This is enabled by the limited pain and swelling, allowing accelerated early phase rehabilitation [23].

### Clinical and functional evaluation

Patients were followed up in the outpatient clinic until 6 months postoperatively. All patients were evaluated through manual clinical examination using Lachman and pivot shift tests. No further testing was performed at that time.

Patients were evaluated prospectively using the Surgical Outcome System (SOS, Arthrex, Naples, FL, USA). SOS is a web-based tool which sends questionnaires and Patient-Reported Outcome Measures (PROMs) by e-mail at prescheduled time-points, after informed consent was given by the patient preoperatively. Prior to introducing the SOS system and analyzing the prospective follow-up data, permission was sought from the local medical ethics committee.

The PROMs collected were the Knee Injury and Osteoarthritis Outcome Score (KOOS) which is a validated outcome score for patients following ACL surgery, the Visual Analogue Pain Scale (VAS-pain) which is a validated scoring system for pain around the knee, the Veterans RAND 12-Item Health Survey (VR-12) which is an established scoring system with widespread use to assess a patients physical and psychological health status and the Marx Activity Scale which measures activity levels of patients and is important in this patient population [9, 36, 39, 40]. These data were collected preoperatively and at 12, 24 and 60 months postoperatively. In addition, a standard questionnaire was completed to ask the patients who did not have any further surgery on the ipsilateral knee about their overall satisfaction with regards to reducing pain, improving movement, resuming normal function and resuming sport. All of the patients were also contacted by email/telephone at the time of this analysis to collect data about any complications.

### Statistical analysis

Descriptive statistics were calculated to summarise the demographics and clinical characteristics and described with means and standard deviations with ranges. Analysis of variance was used to compare the preoperative and postoperative patient-reported outcome measures after exclusion of any patients suffering from a re-rupture and confirmation of normally distributed data using a Shapiro–Wilk test. Tukey–Kramer testing was used to compare all pairs. Additional analyses were performed to compare the re-rupture group and the rest of the patients. Results were considered significant if *p* < 0.05. All analyses were performed with JMP, version 14 (SAS Institute Inc., Cary, NC, USA).

As this was a single cohort study with no control group, a power calculation was not used to guide the study design. Nonetheless, when analysing KOOS score, assuming a mean outcome of 90 based on the literature for ACL reconstruction, with a standard deviation of 15 to detect a clinically meaningful difference of 8% would require a sample of 28 patients, for a power of 0.8 and a significance level of 0.05.

## Results

All patients were found to have a stable knee on manual clinical examination (Grade 1A Lachman and Grade 0 pivot shift tests) when reviewed in the outpatient clinic 6 months postoperatively by the senior author. No further clinical testing was performed. PROMS data were then used to assess the longer term outcomes of these patients.

**Fig. 1:**
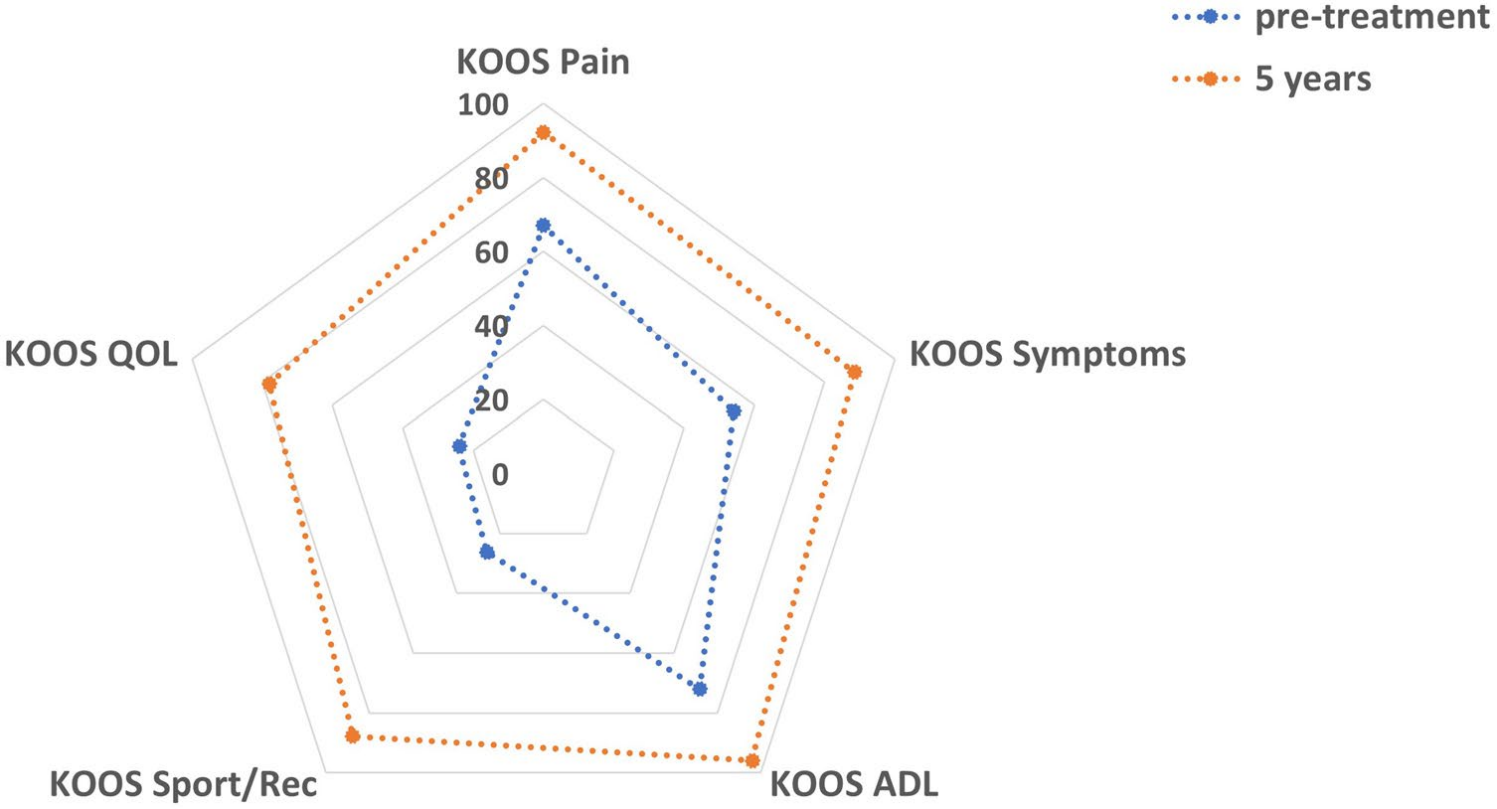
Spider chart demonstrating significant improvements at 5-year follow-up in all sub-sections of the KOOS

The mean KOOS at 5 years was 88.5 (SD 13.8) which improved significantly from 48.7 (SD 18.3) preoperatively (*p* < 0.0001). All subscales of the KOOS demonstrated significant improvements at 5-year follow-up (*p* < 0.0001) as outlined in Fig. 1. No significant differences were seen between the other postoperative time intervals.

The VAS for pain decreased significantly from 2.3 (SD 1.7) preoperatively to 1.0 (SD 1.5) at 5-year follow-up. No significant differences were seen between the other postoperative time intervals.

The VR-12 physical score was 35.9 (SD 10.3) preoperatively and increased significantly to 52.4 (SD 5.9) at 5-year follow-up (*p* < 0.0001). The VR-12 mental score was 54.3 preoperatively and there was minimal change to 53.9 at 5-year follow-up (n.s.). No significant differences were seen between the different postoperative time intervals.

The Marx activity scale decreased significantly from 12.4 (SD 3.4) pre-injury to 7.3 (SD 5.2) at 5-year follow-up (*p* = 0.02). There was very little change between 1 year, 2 years and 5 years postoperatively. The overall decrease in the Marx activity scale postoperatively has also been reported for patients undergoing ACL reconstruction [37, 44].

**Fig. 2:**
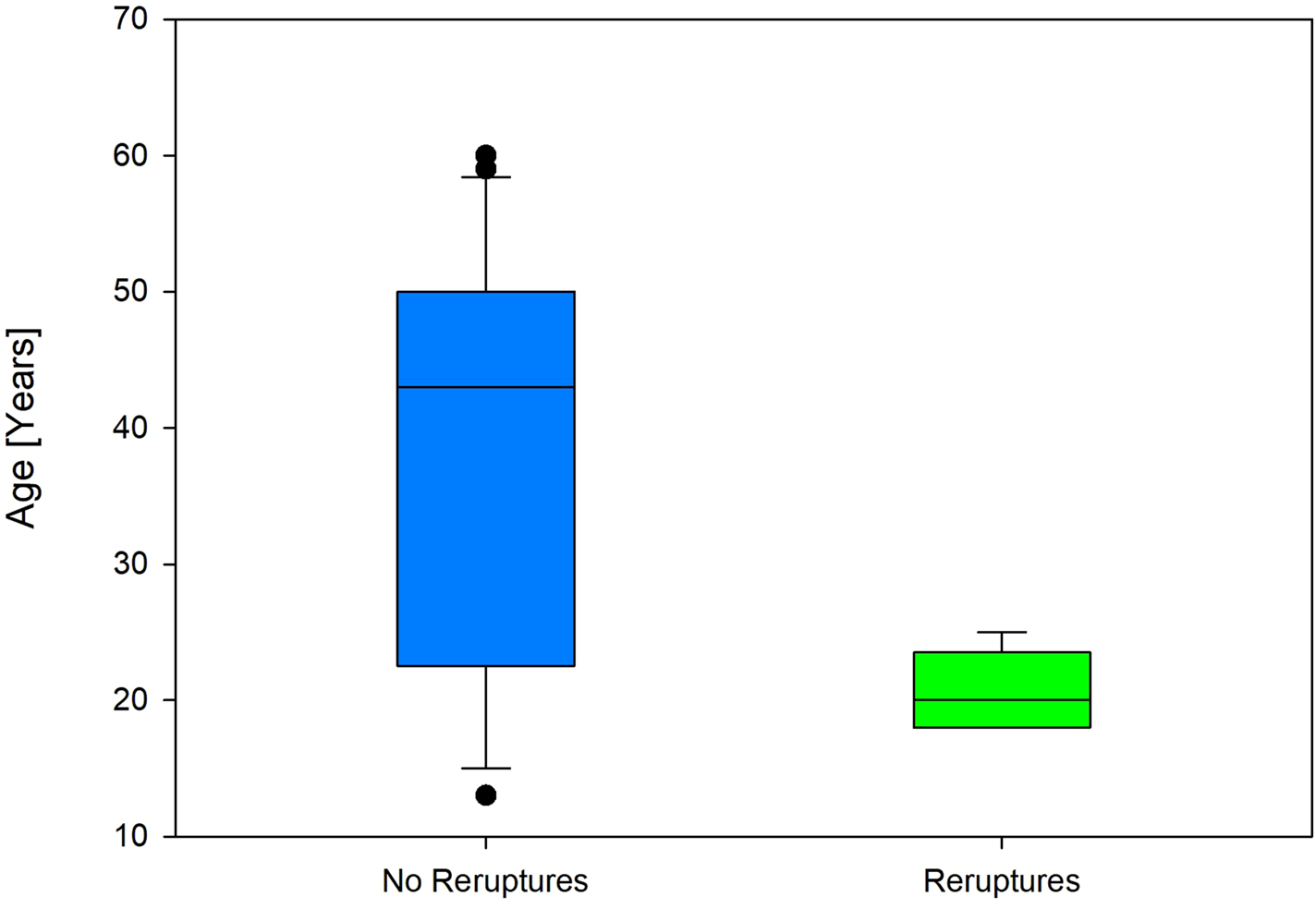
Graph demonstrating the significant differences in age between the re-rupture group and the rest of the patients

Six patients had suffered a re-rupture (17.6%) at the time of this analysis following a significant trauma during sport. All of these patients underwent a standard ACL reconstruction for their revision surgery and have had no issues since then. Three patients (8.8%) underwent secondary meniscal surgery (two partial medial meniscectomies and one partial lateral meniscectomy) following new injuries playing sport. No further complications or further surgery on the knee were reported. The six patients in the re-rupture group were found to be significantly younger (Mean age, 20.7 years) than the rest of the patients as summarized in Fig. 2 (*p* = 0.017). No significant differences were found with gender.

**Fig. 3:**
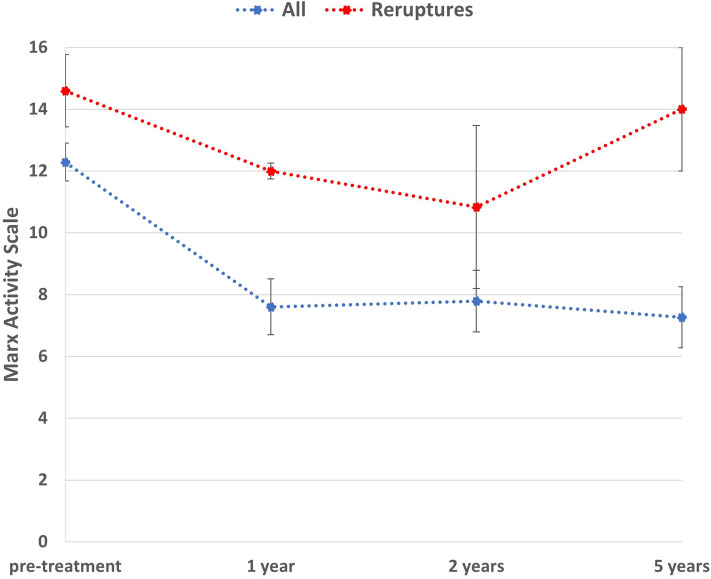
Line graph demonstrating an overall decrease in the Marx activity score and also a significant difference between the 2 groups

In addition, there was a significant difference in the pre-injury Marx Activity Scale between the re-rupture group (14.6) and the other patients (12.3) as outlined in Fig. 3 (*p* = 0.04). Furthermore, Fig. 3 demonstrates the return to normal activity of the patients who have undergone a routine ACL reconstruction as their revision surgery.

## Discussion

The most important findings of the present study were the satisfactory patient-reported outcome measures in 82.4% of patients undergoing ACL repair with suture tape augmentation for acute proximal ACL ruptures at 5-year follow-up. These patients have avoided the need for ACL reconstruction and its associated graft site morbidity and loss of proprioceptive fibers from the native ACL. These outcomes are better than the failure rates of 25–53% associated with primary repairs of the ACL in the 1970s and 1980s which was one of the reasons behind ACL reconstruction becoming the gold standard surgical option for ACL ruptures [15, 18, 33]. However, there are no historical PROMs data for direct comparison. The careful selection of patients undergoing this procedure and the older population of our study group may be contributory factors to the improvements. Nevertheless, the outcomes of the present study are similar to that described for meniscal repair surgery where more than 80% of patients do not require further surgery [19]. Importantly, the tunnels associated with the technique in this study are situated in the same position as the larger tunnels used for hamstring or patellar tendon autografts in ACL reconstruction. As a result, the six failures of our ACL repair technique have had a routine primary ACL reconstruction using autograft without compromise of the knee joint and the additional complications associated with revision surgery [31]. In addition, there was no evidence of synovitis, erosions or cyst formation on further imaging or at the time of revision surgery. This addresses a major concern and highlights the difference between the internal bracing technique used in this study and traditional synthetic grafts [45].

Two-year outcomes for ACL repair with suture tape augmentation in 42 patients undergoing surgery for an acute proximal ACL rupture have previously been described [24]. This paper demonstrated good patient-reported outcome measures with a re-rupture rate of 4.8%. Jonkergouw et al. recently published results of 56 patients with 2-year follow-up with the latter 27 patients having additional suture tape augmentation inserted [26]. This paper showed good objective and subjective outcomes at follow-up which adds to their previously published evidence in this area [10–12]. On the other hand, Gagliardi et al. recently reported high failure rates with internal bracing; however, the mean age of the small cohort of patients undergoing ACL repair was 13 years [21]. As far as we are aware, there are no studies in the literature with the 5-year follow-up, we have described for ACL repair with suture tape augmentation.

Survival rates in the present study are similar to the 5-year outcomes of the dynamic intraligamentary stabilization ACL repair technique (80%), albeit that was a much smaller cohort [13]. Our finding of increased failure rates in young and more active patients are not surprising given these findings have been reported in registry data for several years in relation to traditional ACL reconstructions [2, 3, 17, 22, 27, 35, 43, 50]. Additionally, the overall decrease in the Marx activity scale postoperatively has previously been reported for patients undergoing ACL reconstruction [37, 44].

Nevertheless, the patients in this study underwent an isolated ACL repair with suture tape augmentation between 2011–2014 which is around the time when the anterolateral ligament (ALL) was being rediscovered [7]. Many of the patients in this cohort, in particular those who suffered from a re-rupture could have been deemed high risk and may have benefited from an additional ALL repair with suture tape augmentation to provide rotational stability [25]. Current literature suggests that a combined ACL reconstruction and ALL procedure is the treatment of choice for high-risk patients [38].

There are several limitations associated with this study, namely the lack of clinical testing and radiological assessment at 5 years which is a major limitation. Furthermore, the mean age in this cohort was 37.8 years and it could be these older patients have not put the extra demand on the ACL that a younger patient often does, which is also major limitation. In addition, no comparisons can be made to ACL reconstruction procedures as there was no randomisation and all of the patients within the inclusion criteria underwent ACL repair with suture tape augmentation.

## Conclusion

Primary repair with suture tape augmentation for proximal ACL tears demonstrates satisfactory outcomes in 28 patients (82.4%) at a minimum of 5 years following their surgery. Six patients sustained a re-rupture, all of whom were subsequently treated with a standard ACL reconstruction with no complications thereafter. These patients were significantly younger and had higher initial Marx activity scores.
